# Left atrial structure and functional quantitation using cardiovascular magnetic resonance and multimodality tissue tracking: validation and reproducibility assessment

**DOI:** 10.1186/s12968-015-0152-y

**Published:** 2015-07-01

**Authors:** Mytra Zareian, Luisa Ciuffo, Mohammadali Habibi, Anders Opdahl, Elzbieta H. Chamera, Colin O. Wu, David A. Bluemke, João A. C. Lima, Bharath Ambale Venkatesh

**Affiliations:** MR 110, Radiology, Johns Hopkins University, Baltimore, MD 21287 USA; Oslo University Hospital and University of Oslo, Oslo, Norway; National Institutes of Health, Bethesda, MD USA

**Keywords:** Left atrial function, Tissue tracking, Left atrial strain, Reproducibility, Cardiovascular magnetic resonance

## Abstract

**Background:**

Left atrium (LA) strain, volume and function are important markers of cardiovascular disease and myocardial impairment. We aimed to assess the accuracy of LA biplane volume and function measured by Multimodality Tissue Tracking (MTT). Also we assessed the inter-study reproducibility for cardiovascular magnetic resonance (CMR) derived LA volume and function parameters.

**Methods:**

Thirty subjects (mean age: 71.3 ± 8.7, 87 % male) including twenty subjects with cardiovascular events and ten healthy subjects, with CMR were evaluated in the Multi-Ethnic Study of Atherosclerosis (MESA). LA volumes were computed by the modified biplane method from 2- and 4-chamber projections and the Simpson’s method from short-axis slices using both methods - manual and semi-automated delineation using MTT. LA total, active and passive ejection fractions were calculated. Pearson’s correlation and Bland-Altman analysis were used to compare the measurements. In a second sample of 25 subjects (age: 65.7 ± 7.1, 72 % males) inter study, intra and inter reader reliability analysis was performed. The intra-class correlation coefficient (ICC) was evaluated.

**Results:**

Left atrial MTT structural and functional parameters were not different from manual delineation, yet image analysis was only half as time consuming on average with MTT. Maximal volume MTT was not different between the Simpson’s and Biplane methods, functional parameters, however were different. MTT allowed us to measure multiple LA parameters with good-excellent (ICC; 0.88– 0.98, *p* < 0.001) intra-and inter reader reproducibility and fair-good (ICC; 0.44–0.82, *p* < 0.05–0.001) inter study reproducibility.

**Conclusions:**

MTT derived LA biplane volume and function is accurate and reproducible and is suited for use in longitudinal studies.

## Background

Left atrium (LA) enlargement is associated with adverse cardiovascular outcomes [1, 2]. Studies have reported the relationship between increased LA size and the incidence of heart failure (HF), atrial fibrillation (AF), stroke and risk of overall mortality after myocardial infarction (MI) [1–4]. Furthermore, LA function is believed to be a dynamic marker of both the severity and chronicity of diastolic LV dysfunction [3, 5].

The American Society of Echocardiography recommends the quantification of LA by 2-D echocardiography using either the biplane area length method or the method of discs [1, 6]. However, 2D and 3D echo usually underestimate LA size and volumes as compared to cardiovascular magnetic resonance (CMR) and MSCT [1, 7]. The higher spatial resolution and non-invasiveness afforded by CMR has made it a preferred method for the assessment of cardiac anatomy, dimensions, function and mass [8]. The standard short-axis method of measuring left atrial volume and ejection fraction is very time-consuming both in terms of acquisition of additional slices as well as additional analysis time [8, 7]. While global cardiac function is more often reported and used as clinical parameter of cardiac status, some studies have demonstrated that regional myocardial strain may be more sensitive in detecting early myocardial dysfunction [9].

The aims of this study are (i) to validate feature tracking using the Multimodality Tissue Tracking (MTT) software for CMR for quantifying LA volumes and functional (global and regional) parameters; (ii) to compare the biplane method with the Simpson’s method; (iii) to establish inter-study reproducibility of strain and function measured from the bi-plane method.

## Methods

### Study population

This ancillary study was designed in the Multi-Ethnic Study of Atherosclerosis (MESA). MESA, which was initiated in 2000, is a prospective observational multi-center cohort study [10]. Participant’s ages ranged 45–84 years and all were asymptomatic of clinical CVD at enrollment. The institutional review boards of all centers approved this study and informed consent was obtained from every participant. More detailed information about the MESA study goals and methods can be found elsewhere [10].

**Fig. 1:**
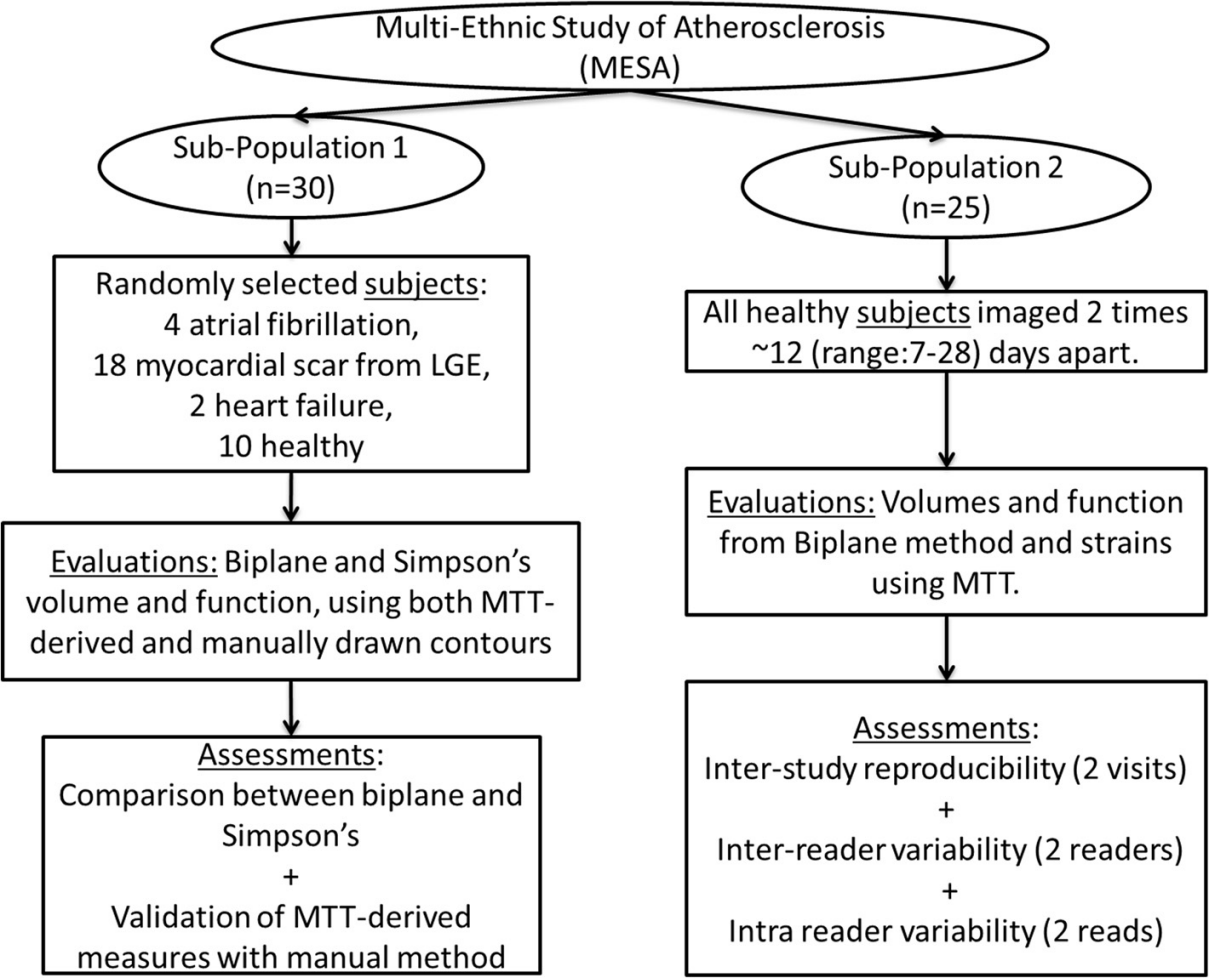
Scheme of the study design. Illustration also shows the measurements that were evaluated as well as the assessments/aims associated with each set of subjects

For this study, 2 sets of subjects were chosen. Please see Fig. 1 for a detailed illustration of the 2 sets of subjects and specific aims associated with each population.

i. Population 1 consisted of thirty selected subjects (mean age: 71.3 ± 8.7, 87 % male) including twenty subjects with prior cardiovascular events (4 atrial fibrillation, 18 myocardial scar from late gadolinium enhancement, 2 heart failure) and ten healthy subjects, with CMR imaging were evaluated from the MESA 10-year follow-up exam (2010 to 2012). The twenty subjects with prior cardiovascular events were chosen randomly from a sample of 233 participants with atrial fibrillation, myocardial scar or heart failure – all factors that have been associated with modified structure and reduced LA function; while the 10 healthy subjects were chosen from 2634 participants with no prior cardiovascular disease. The study was so designed to compare performance across the complete range of expected LA structure and function. In this first sample, a comparison between biplane and Simpson’s method; and the validation of MTT against the manual method (using QMass Medis, Leiden, Netherlands) was performed.
ii. The second sample, population 2, was composed of 25 subjects chosen randomly who agreed to be part of the reproducibility study performed at Johns Hopkins University. These subjects were enrolled between 2008 and 2010 and had a baseline and follow-up exam 12 ± 7 days (range, 7–28 days) days apart. On this sample we established inter study, intra and inter reader reliability.

### CMR protocol

All participants underwent CMR using a 1.5 T scanner (Avanto; Siemens Medical Systems, Erlangen, Germany) with a gradient strength of 45 mT/m, slew rate of 200 Tm^−1^/s. The cine images included coverage of the entire LV and LA using short-axis slices, one 2-chamber slice and one 4-chamber view scanned by steady-state free precession sequences (SSFP) with the following parameters: Slice thickness: 8 mm; Gap: 2 mm; Temporal resolution: 35 ms (30 frames); Matrix: 256×256 and Field of view: 360 × 360 mm.

### CMR analysis

#### Multimodality tissue tracking

**Fig. 2:**
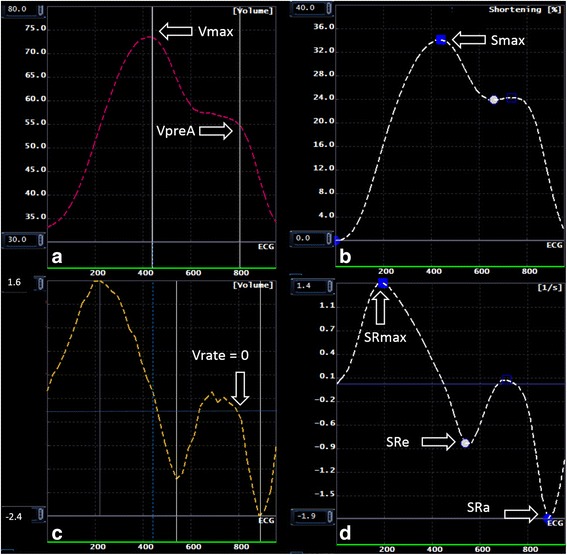
Multimodality Tissue Tracking, volume analysis (Panel **a**), volume rate analysis (Panel **b**), strain analysis (Panel **c**) and strain rate analysis (panel **d**). Green line: End-systole. White line: Point where volume-rate curve: 0; Corresponding VpreA in the volume curve

Multimodality Tissue tracking software (MTT; version 6.0, Toshiba, Japan) is an automated frame-to frame template matching software [11, 12]. Initially, an experienced operator defines the LA endocardial and epicardial borders at the reference frame - ventricular end-systolic frame identified just before mitral valve opening, when the LA is at its biggest dimension (Fig. 2). The confluence of the pulmonary veins and LA appendage are not included in the segmentation.

The software then propagated these borders across the cardiac cycle automatically using a template matching algorithm. The software recorded a characteristic pixel pattern of each 10 × 10 mm square area in the reference frame; an area with identical pixel pattern was recognized in the next frame that maximized the similarity evaluated by cross-correlation between the square areas. This procedure was repeated for all pixels in each image and for each frame to track the borders throughout the whole cardiac cycle [13]. Finally, the operator verified the quality of the tracking generated by the software.

MTT was used in untagged long-axis 2-chamber and 4-chamber projections to obtain:

- Maximum LA volume (V_max_): LA volume at end-systole, immediately before mitral valve opening.
- Minimum LA volume (V_min_): LA volume at end-diastole, immediately before mitral valve closure.
- Pre-atrial contraction volume (V_preA_): LA volume at onset of the P-wave on ECG.
- Strain rate at maximum (S_max_): Peak global longitudinal strain. Indirect measurement of atrial relaxation during LV systole.
- LA strain rate at maximum (SR_max_): Time derivate peak strain rate during ventricular systole.
- Early LA diastolic peak (SRe): Time derivate first (ventricular) diastolic LA strain peak.
- Atria contraction peak (SRa): Time derivate maximum strain measured at atrial contraction. Second (ventricular) diastolic LA strain peak.

**Fig. 3:**
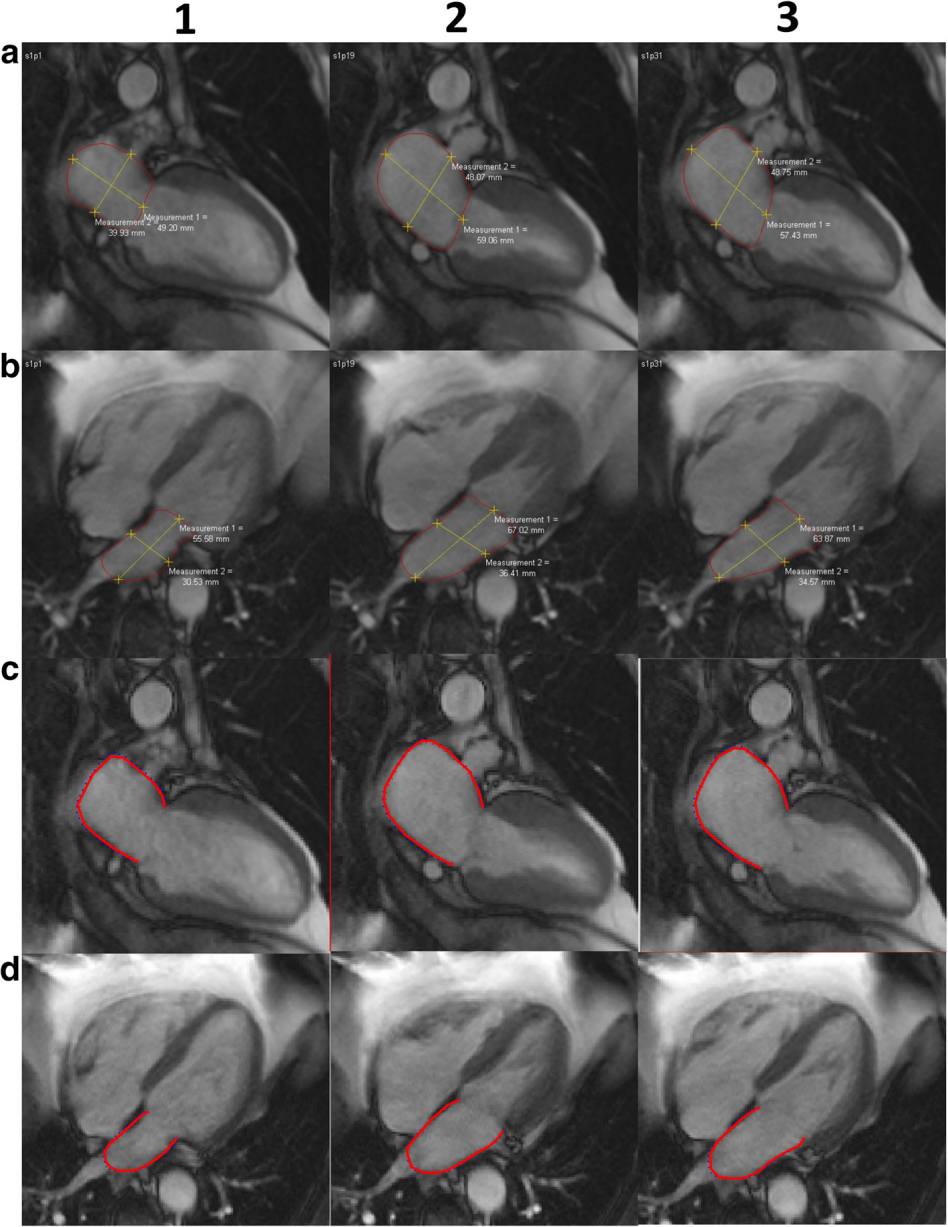
Biplane method. Manual delineation requires manual drawing of the endocardial contours in (1) end-diastolic, (2) end-systolic and (3) pre-atrial kick phase, separately for 2 (**a**) and 4 (**b**) chamber projections. Corresponding contours using Multimodality Tissue Tracking software are shown at end-diastole (**c**) and endsystole (**d**). The contours were drawn at left-ventricular end-systole (the point of largest LA enlargement) and were propagated by the software throughout the cardiac cycle

All the above parameters were obtained from strain, strain rate and volume curves from MTT (Fig. 3). LA performs three different functions during the cardiac cycle: 1) acts as a reservoir during LV systole; 2) acts as a conduit in early LV diastole; 3) acts as an active pump during late LV diastole [1]. Taking this information in consideration, we performed the measurement of pre-atrial contraction volume at the point where the rate of change of atrial volume was closest to zero, at this point the atria acts as a conduit, thus, only minor changes in volumes can be visualized in the LA, representing the transition between atrial conduit phase and atrial contraction phase (Fig. 3) [14]. Left atrial ejection fraction (LAEF %) was calculated as: (Vmax-Vmin)/Vmax × 100; Left atrial passive ejection fraction (LAPEF %): (Vmax-VpreA)/Vmax × 100 and Left atrial active ejection fraction (LAAEF %): (VpreA-Vmin)/VpreA × 100.

### The biplane area-length method

The formula on which the biplane method is based on is as follows: LA volume = (0.848 * area_4chamber_ * area_2chamber_)/(length_2chamber_ + length_4chamber_)/2 [6] (Fig. 2). The LA appendage and the confluence of the pulmonary veins at its ostium are excluded. The Simpson’s method essentially is the summation of the cross-sectional areas of each slice accounting for slice thickness and the interval between slices from short axis views. Volumes were calculated at the end-diastolic, end-systolic and pre-atrial phases, all the phases were determined based on visual inspection of the chamber through the cycle in the manual delineation (requires drawing contours at each time) method using QMass (Medis, Leiden, Netherlands).

### MTT reproducibility

Intra reader MTT reproducibility was established by one reader who performed analysis of the studies twice using MTT software to generate LA functional and structural parameters, the interval between the two analyses were at least 7 days. Inter reader reproducibility was assessed by two readers who analyzed the same cases using MTT software to generate LA data. The second reader was blinded to the results of the first reader.

### Statistical analysis

Data are presented as mean ± standard deviation (SD) for continuous variables and as percentages for categorical variables. A paired student’s two-tailed *t* test was used to determine significant differences between the two sets of methods and software’s. Linear regression analysis and Pearson’s correlation were also used to examine the relationship between the two methods. Pearson’s correlation coefficient was scored as follows: poor correlation, 0; slight, 0.01–0.20; fair, 0.21–0.40; moderate, 0.41–0.60; good, 0.61–0.80, and excellent, 0.81–1.00 correlation.

For intra-and inter-observer reproducibility and inter study reproducibility a Bland-Altman analysis and Passing-Bablok regression were performed [15, 16]. Moreover the intra-class correlation coefficient (ICC) with a two way random model (ICC, <0.40, poor; ICC >0.40–0.75, fair to good; and ICC >0.75, excellent agreement) was evaluated. For inter-study reproducibility, Absolute measurement error was estimated by the standard error of the measurement (SEM) and smallest detectable change (SDC) [17]. We performed the calculations using the following formulas: SEM = SD × √ (1-ICC) and SDC = 1.96 × SEM × √2. SDC was established taking in consideration 95 % confidence interval (1.96). Statistical analysis was done using SPSS version 20 (SPSS Inc., Chicago). MedCalc (MedCalc Software version 13.2.2.0, Mariakerke, Belgium) was used to perform Bland-Altman plots and regressions graphics.

## Results

The participant characteristics for both samples are show in Table 1. The first sample (population 1) was composed by individuals with the following characteristics; mean age 71.3 ± 8.7 years and 86.7 % were men. A larger proportion was Caucasian (53.3 %) and African-American (46.7 %) than in the overall population of participants at the MESA Exam 5. Among these subjects 23 % had diabetes mellitus, 33 % had a diagnosis of hypertension. One case was excluded from the first sample because of MRI technical limitations (short axis image did not cover the entire LA).

**Table 1:** Participant characteristics

Parameter	Sample 1 (*n* = 30)	Sample 2 (*n* = 22)	*p*
Age (years)	71.3 ± 8.7	65.7 ± 7.1	0.01
Gender, male (%)	86.7	71.4	
Ethnicity			
African-American (%)	46.7	33.3	
Caucasian (%)	53.3	66.6	
BMI (kg/m2)	30.1 ± 4.8	29.8 ± 5.4	0.60
SBP (mmHg)	123.8 ± 15.3	123.3 ± 18.5	0.92
DBP (mmHg)	65.3 ± 11.2	72.7 ± 10.3	0.33
Heart rate (bpm)	65.8 ± 12.6	60.4 ± 11.1	0.13
Antihypertensive medication (%)	67		
DM/IFG (%)	50	50	
Total cholesterol	167.5 ± 32.4	197.1 ± 41.5	<0.05

Results are reported as mean ± standard deviation. Patient characteristics for participants in: sample 1- the validation study, and sample 2- the reproducibility study

Abbreviations: *BMI* Body mass index, *SBP* Systolic blood pressure, *DBP* Diastolic blood pressure, *DM* Diabetes mellitus, *IFG* Impaired fasting glucose

The second sample (population 2) was composed by individuals with the following characteristics; mean age 66.4 ± 7.15 years and 71.4 % were men. Of these, 28 % had diabetes mellitus, 56 % had a diagnosis of hypertension. Three subjects were excluded due to: poor orientation of the four Chamber View and significant image artifacts.

### Population 1

#### MTT validation

**Fig. 4:**
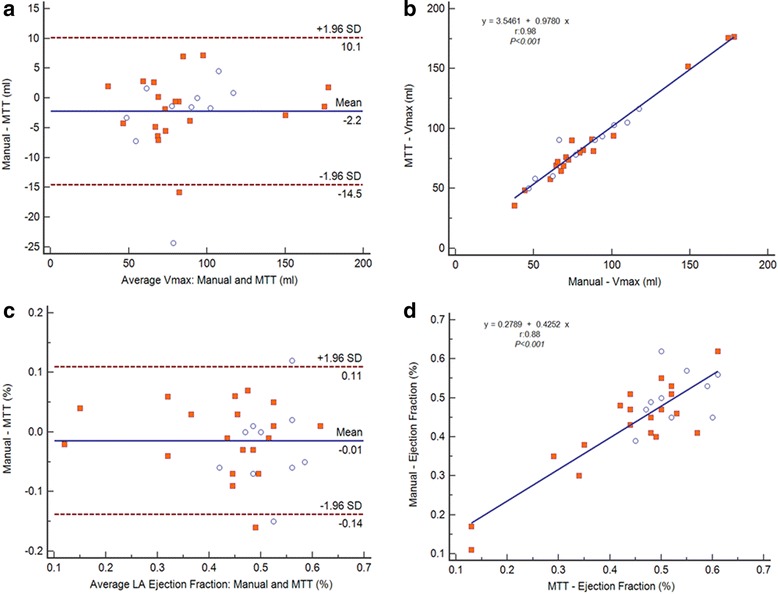
**a**–**d** Linear regressions (right) and Bland-Altman (left) plots analysis. The Pearson’s correlation coefficient (r) and SD = Standard deviation. LA Vmax: Manual vs. MTT (**a** - **b**). LA Ejection Fraction: Manual vs. MTT (**c** - **d**)

Table 2 shows comparison between the Manual vs. MTT derived Biplane LA Volume and Global Function measures. No significant differences between the manual and MTT-derived bi-plane measures were found for any of the parameters analyzed. Moreover, they had good-to-excellent correlation (r: 0.83–0.98, *p* < 0.001) and agreement as shown in the Bland-Altman plots for all variables (Figs. 4a-d); except for LAPEF which demonstrated the lowest correlation (r:0.53, *p* < 0.001) and less agreement (Mean Difference ± SD of difference). Image analysis was less time consuming on average with MTT (Simpson’s: MTT vs. manual: 3:10 min vs. 7:23 min; Biplane, MTT vs. manual: 1:30 min vs. 8:28 min).

**Table 2:** Comparison between the manual vs. MTT derived biplane volume and global function measures (n = 29)

LA parameter	MTT	Manual	*P*	*r*	*P*
Vmax (ml)	86.5 ± 33	84.4 ± 34	0.08	0.98	<0.001
LAEF (%)	0.45 ± 0.13	0.44 ± 0.12	0.21	0.88	<0.001
LAPEF (%)	0.17 ± 0.05	0.16 ± 0.08	0.67	0.57	<0.001
LAAEF (%)	0.37 ± 0.1	0.36 ± 0.09	0.21	0.83	<0.001

Results are reported as mean ± standard deviation

Abbreviations: *Vmax* left atrial maximal volume, *LAEF* left atrial total ejection fraction, *LAPEF* left atrial passive ejection fraction, *LAAEF* left atrial active ejection fraction, *r* Pearson coefficient

### Comparison of Simpson’s vs. biplane methods assessed by MTT

**Fig. 5:**
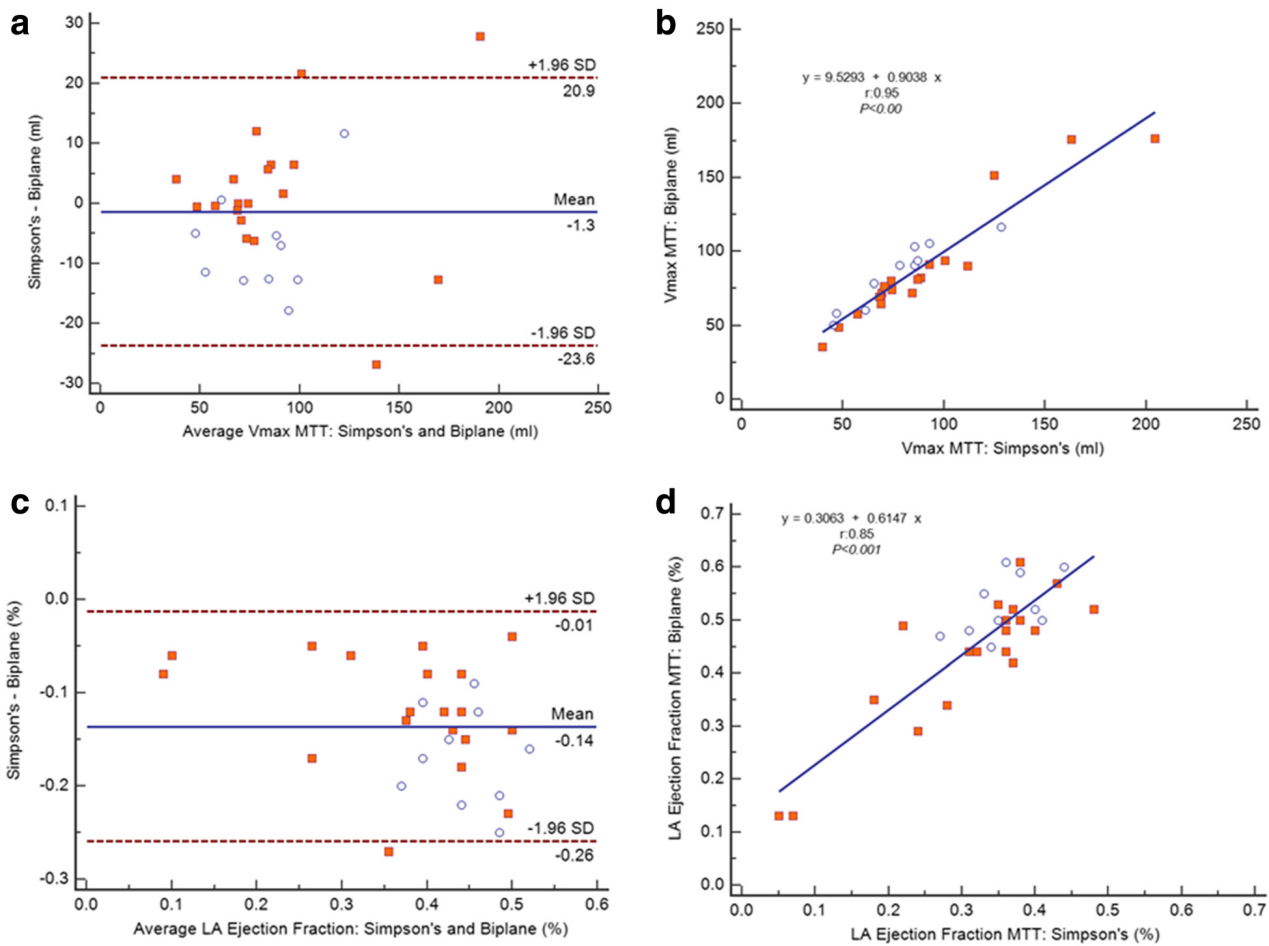
**a**–**d** Linear regressions and Bland-Altman plots analysis. The Pearson’s correlation coefficient (r) and SD = Standard deviation. MTT: Biplane area-length vs. Simpson’s (**a** - **b**); MTT: LA ejection fraction derived Biplane area-length vs. LA ejection fraction derived Simpson’s (**c** - **d**)

LA maximum volumes obtained from MTT method were not significantly different between Simpson’s and biplane methods: 85.2 ± 35.2 vs. 86.5 ± 33.6 (Fig. 5). However, there was a statistically significant difference between all the functional global parameters: LAEF (Biplane: 0.46 ± 0.2, Simpson: 0.33 ± 0.10, *p* <0.001), LAPEF (Biplane: 0.18 ± 0.05, Simpson: 0.11 ± 0.04, *p* <0.001) and LAAEF (Biplane: 0.37 ± 0.07, Simpson: 0.27 ± 0.06, *p* <0.001). Functional measurements established by Simpson’s method were systematically lower. The same trend was found in the analysis of biplane vs. Simpson’s performed by manual method (Table 3).

**Table 3:** Comparison between the Simpson’s method vs. Biplane method using MTT and Manual (n = 29)

Manual	Biplane	Simpson’s	*P*	*r*	*P*
Vmax (ml)	84.3 ± 34.6	88.1 ± 35.2	0.08	0.95	<0.001
LAEF	0.44 ± 0.1	0.34 ± 0.1	<0.001	0.88	<0.001
LAPEF	0.21 ± 0.22	0.17 ± 0.23	<0.001	0.70	<0.001
LAAEF	0.36 ± 0.08	0.28 ± 0.07	<0.001	0.70	<0.001
MTT	Biplane	Simpson’s	*P*	*r*	*P*
Vmax (ml)	86.5 ± 33.6	85.2 ± 35.2	0.53	0.95	<0.001
LAEF	0.46 ± 0.1	0.33 ± 0.10	<0.001	0.85	<0.001
LAPEF	0.22 ± 0.21	0.16 ± 0.23	<0.001	0.57	0.002
LAAEF	0.38 ± 0.09	0.27 ± 0.06	<0.001	0.61	<0.001

Results are reported as mean ± standard deviation

Abbreviations: *Vmax* left atrial maximal volume, *LAEF* left atrial total ejection fraction, *LAPEF* left atrial passive ejection fraction, *LAAEF* left atrial active ejection fraction, *r* Pearson coefficient

### Population 2

#### MTT inter, intra-reader and test-retest reproducibility

**Fig. 6:**
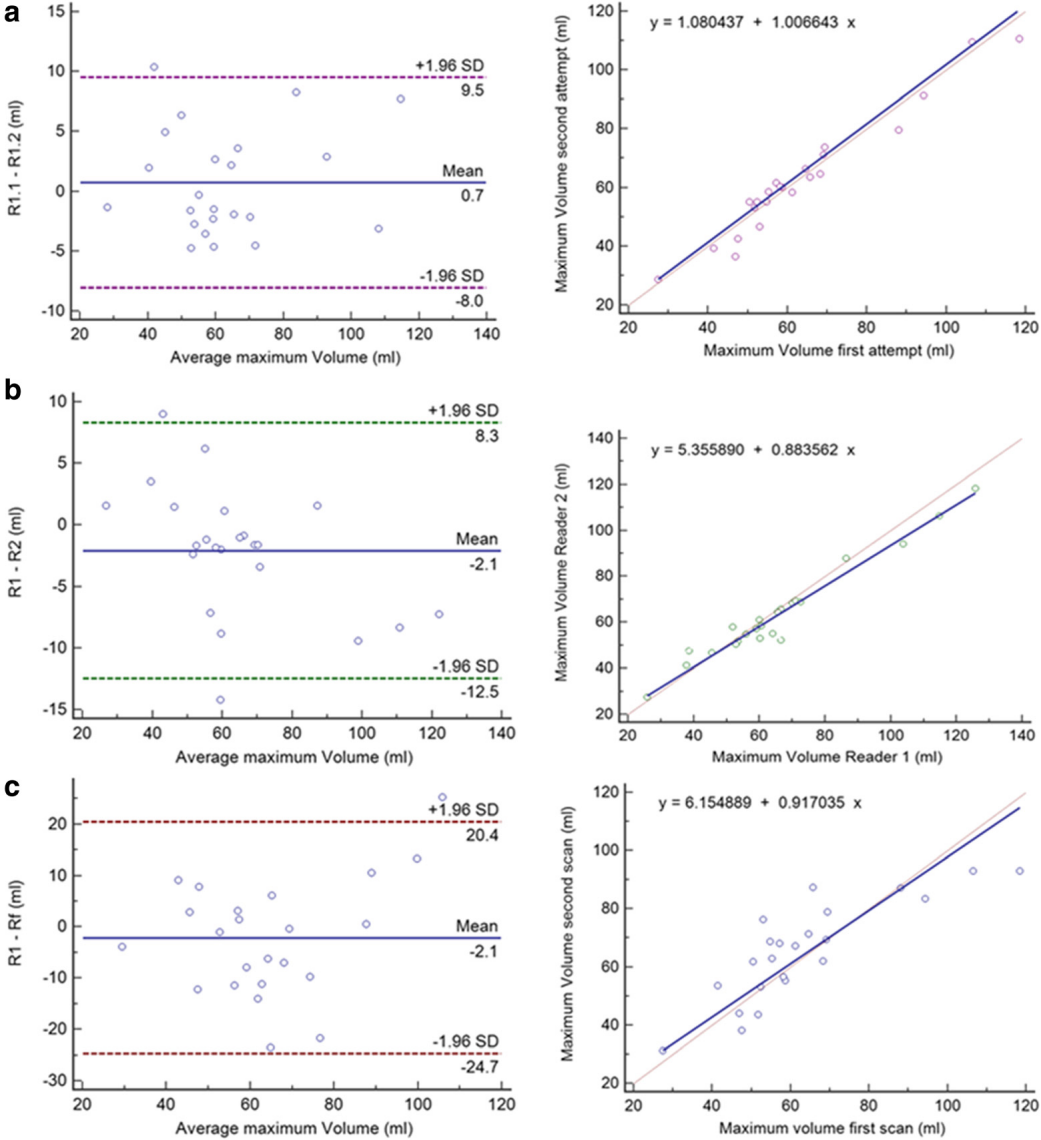
Intraobserver (**a**), interobserver (**b**) and inter study variability (**c**) of LA maximum Volume: Bland Altman plot (left) and Passing-Bablok regression (right), SD = Standard deviation

**Fig. 7:**
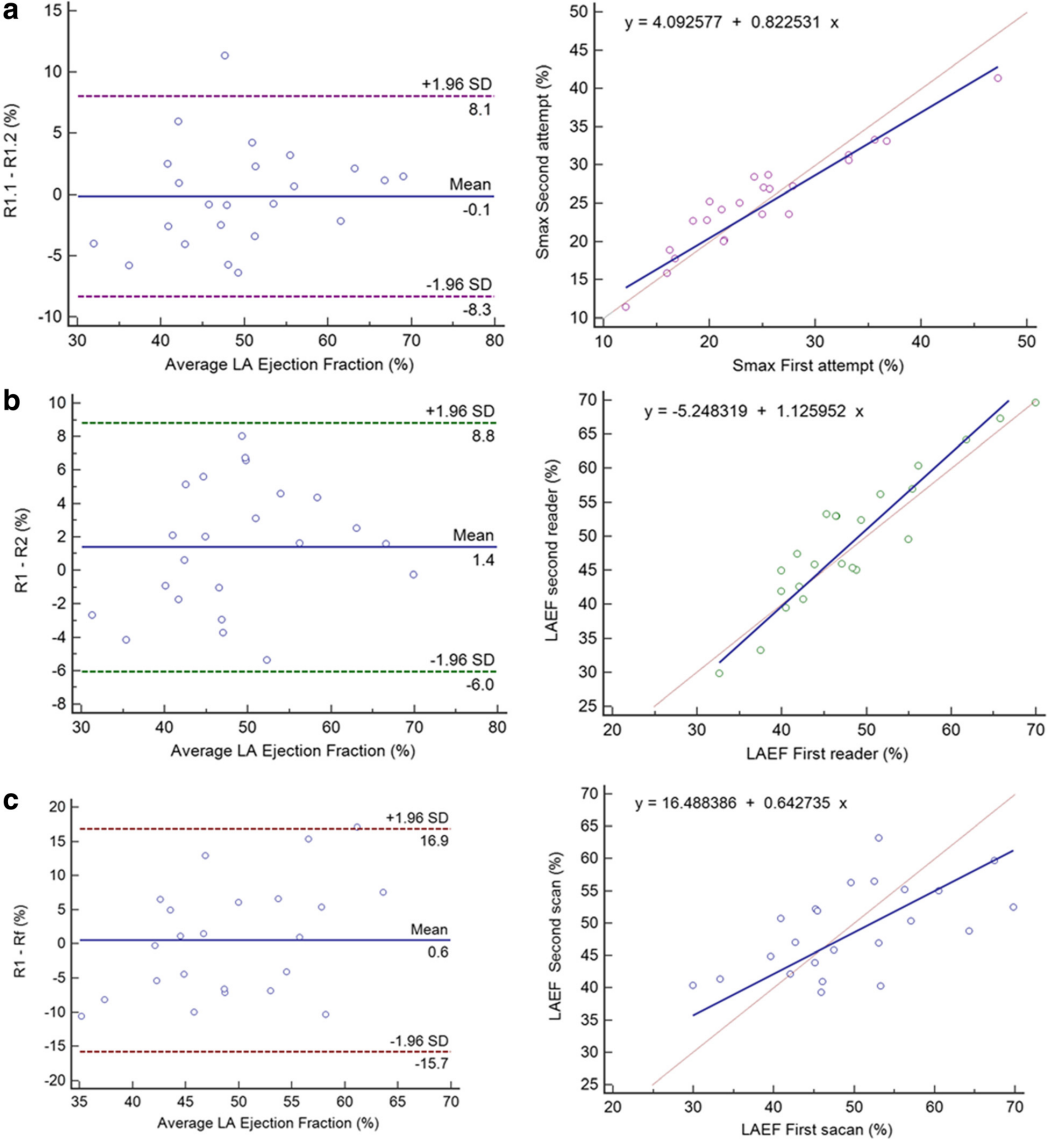
Intraobserver (**a**), interobserver (**b**) and inter study variability (**c**) of LA Ejection Fraction: Bland Altman plot (left) and Passing-Bablok regression (right), SD = Standard deviation

**Fig. 8:**
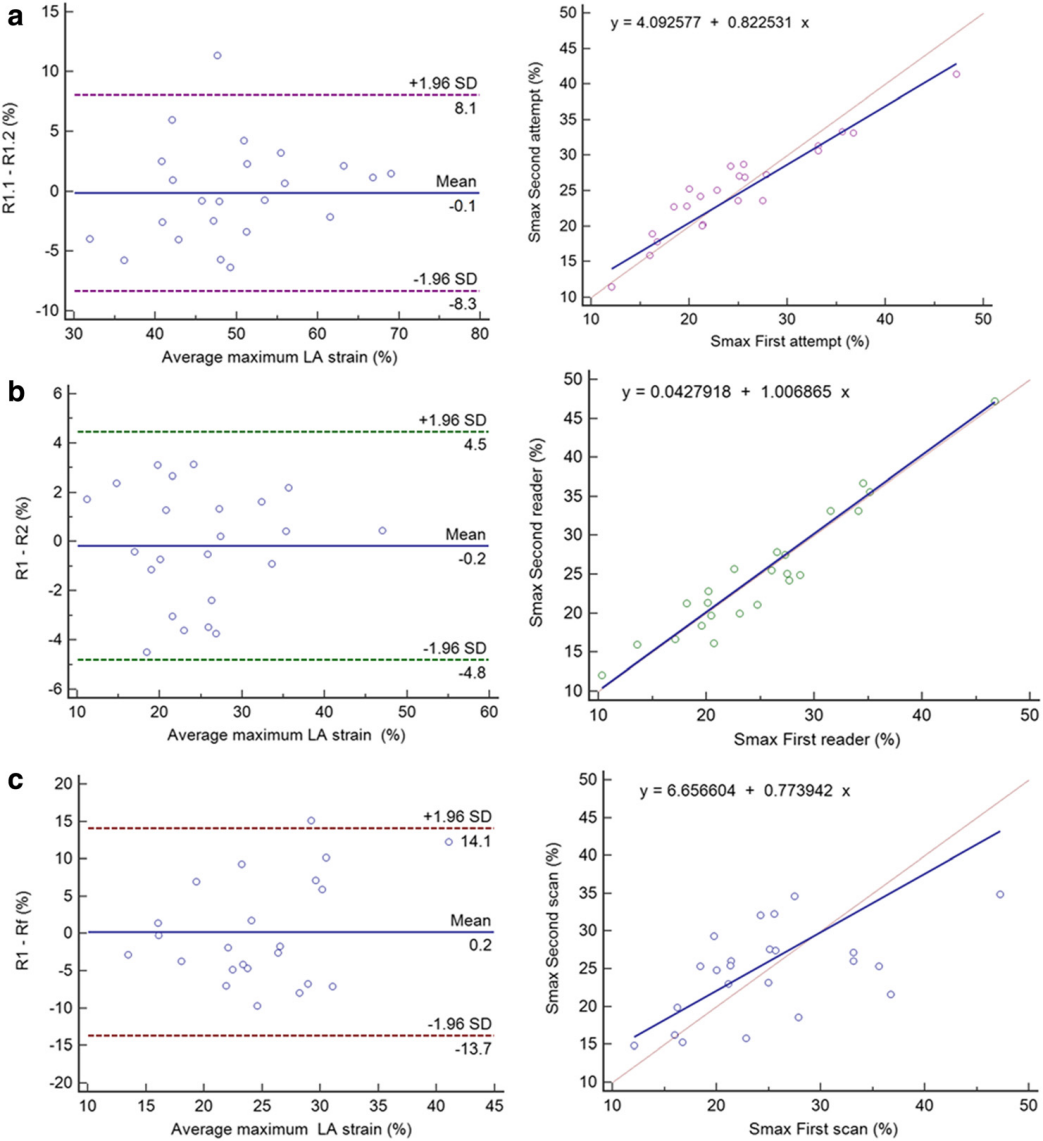
Intraobserver (**a**), interobserver (**b**) and inter study variability (**c**) of LA Smax: Bland Altman plot (left) and Passing-Bablok regression (right), SD = Standard deviation

Inter observer and intra observer variability of LA analysis for the MTT method was assessed in 22 subjects (Table 4, Figs. 6 and 7). All parameters showed excellent intra reader reproducibility (ICC; 0.88–0.98, *p* < 0.001) without significant systematic bias. Except for Sra % (ICC; 0.54, *p* < 0.05) the other parameters showed excellent inter reader reproducibility (ICC; 0.89–0.96, *p* < 0.001). Retest reproducibility (Table 5, Fig. 8) showed fair to good agreement (ICC; 0.44–0.82, *p* < 0.05–0.001) between all parameters.

**Table 4:** MTT Inter- and intra-observer reproducibility for left atrium measurement (n = 22)

	Inter-reader				Intra-reader			
LA parameter	Difference (mean ± SD)	*P*	ICC	*P*	Difference (mean ± SD)	*P*	ICC	*p*
Vmax (ml)	−2.25 ± 5.29	0.07	0.97	<0.001	0.74 ± 4.48	0.44	0.98	<0.001
LAEF (%)	1.40 ± 3.80	0.91	0.92	<0.001	−0.11 ± 4.17	0.90	0.91	<0.001
LAPEF (%)	0.43 ± 4.16	0.62	0.82	<0.001	0.68 ± 3.28	0.33	0.88	<0.001
LAAEF (%)	1.54 ± 5.05	0.16	0.87	<0.001	−0.64 ± 5.08	0.55	0.87	<0.001
Smax (%)	−0.16 ± 2.37	0.75	0.96	<0.001	−0.34 ± 2.96	0.59	0.92	<0.001
SRmax (%/ms)	0.05 ± 0.16	0.13	0.91	<0.001	−0.04 ± 0.15	0.19	0.89	<0.001
SRe (%/ms)	0.009 ± 0.16	0.79	0.93	<0.001	−0.02 ± 0.11	0.50	0.96	<0.001
SRa (%/ms)	−0.12 ± 0.57	0.34	0.59	<0.05	−0.08 ± 0.27	0.16	0.92	<0.001

Results are reported as mean ± standard deviation

Abbreviations: *Vmax* left atrial maximal volume, *LAEF* left atrial total ejection fraction, *LAPEF* left atrial passive ejection fraction, *LAAEF* left atrial active ejection fraction, *Smax* maximum systolic strain, *SRmax* maximum systolic strain rate, *SRe* early-diastolic strain rate, *SRa* atrial diastolic strain rate, *ICC* intra-class correlation coefficient

**Table 5:** MTT test-retest reproducibility for CMR left atrium measurement (n = 22)

LA parameter	Difference (mean ± SD)	*p*	ICC	*p*	SEM	SDC
Vmax (ml)	−2.13 ± 11.52	0.20	0.80	<0.001	5.15	14.28
LAEF (%)	0.58 ± 8.31	0.42	0.54	0.005	5.76	15.95
LAPEF (%)	0.27 ± 6.22	0.99	0.48	0.01	4.49	12.44
LAAEF (%)	0.71 ± 7.77	0.24	0.57	0.002	5.09	14.12
Smax (%)	0.23 ± 7.09	0.84	0.60	0.001	4.49	12.43
SRmax (%/ms)	0.006 ± 0.29	0.72	0.48	0.01	0.21	0.59
SRe (%/ms)	−0.11 ± 0.34	0.16	0.63	<0.001	0.20	0.57
SRa (%/ms)	−0.02 ± 0.59	0.95	0.58	0.002	0.38	1.05

Results are reported as mean ± standard deviation

Abbreviations: *Vmax* left atrial maximal volume, *LAEF* left atrial total ejection fraction, *LAPEF* left atrial passive ejection fraction, *LAAEF* left atrial active ejection fraction, *Smax* maximum systolic strain, *SRmax* maximum systolic strain rate, *SRe* early-diastolic strain rate, *SRa* atrial diastolic strain rate, *ICC* intra-class correlation coefficient, *SEM* standard error of measurement, *SDC* smallest detectable change

## Discussion

The main findings of this study can be summarized as follows: (i) Long axis MTT structural and functional parameters were similar to those from manual delineation; (ii) Maximal volume assessed by MTT was not different between the Simpson’s and Biplane method, functional parameters, however were different. (iii) MTT allowed us to measure multiple LA parameters with good-excellent intra-and inter reader reproducibility and fair-good inter study reproducibility.

LA enlargement is a predictor of poor clinical outcome, especially in patients affected by AF [1–3]. In the clinical setting, volume determinations for LA size are preferred over linear dimensions because of the more accurate assessment of the asymmetric remodeling of the LA chamber [1, 18]. The gold standard method for the assessment of LA volume is the short axis model which is well known but time consuming, thus less used [8, 19, 7]. Our study showed that the more practical and faster assessment of LA maximum volume using biplane area length method had good agreement and it did not identify significantly different maximum volumes when compared with the short–axis based Simpson’s method, these results were similarly to data already presented in other studies in MRI and CT [19–22]. These studies did however not assess LA active and passive function. The differences in function indicate that the changes in LA volume are perhaps, less accurately captured using the bi-plane methods than the Simpson’s method. The error on estimation of LAEF, in both manual and MTT methods, may be consequence of the biplane underestimation of LA minimal volume; thus, overestimation of LAEF, probably due to a more irregular shape of the LA at end of LA systole (Table 3). This bias was seen to be consistent as seen in the Bland-Altman plot for the range of LAEF seen in our study. It is known that the biplane method may be erroneous when long-axis slices acquired are not aligned correctly or when the normal LA shape is distorted under different clinical conditions [23, 7]. Despite these technical issues, LAEF estimation using biplane formula is significantly different in those with infarction [13] and in heart failure [11]. Moreover, other studies have already established the clinical utility of bi-plane LA function in a number of conditions [19, 20, 24, 13, 6]. The validation of MTT against standard manual method did not show any significant differences among structural and functional parameters and showed good-to-excellent correlation. MTT image analysis was less time consuming on average which is crucial for application in a clinical scenario.

Most studies assessing strain using tissue tracking CMR have been restricted to the LV [25–27]. Our results showed a good to excellent level of agreement for the variables analyzed for inter and intra reader reproducibility. The only exception was inter reader analysis of SRa (ICC; 0.54, *p* < 0.05) that represents strain peak during LA contraction. The temporal resolution of ~25–35 ms may not allow accurate capture of the phenomenon, resulting in a lower level of agreement between analyses performed by two different readers. Data from at least one ultrasound intra-reader study showed a similar pattern, SRa was less reliable with an ICC of 0.491 [28].

To the best of our knowledge, this is the first study that performed analysis of LA test-retest- reproducibility of structural and functional parameters using Tissue tracking technique. Our results showed fair to good agreement between all measurements (ICC; 0.44–0.82, *p* < 0.05) and no significant systematic bias was observed. There are multiple factors which could influence the result of retest- reproducibility; technologist variability in performing the examination, radiologist intra-observer variability in each measurement, inter-instrumentation variability due to the utilization of different MR units and biological variability in consequence of patient changing health status between the two examinations. We had an excellent intra- reader agreement; our sample was composed only by individuals who had the exam performed at the same center (Johns Hopkins University, Baltimore) and using the same CMR scanner. Moreover, the short period of time between the two scans 12 ± 7 days (range, 7–28 days) may reduce the contribution of biological variability in LA parameters assessed in this study. The studies were performed by technicians who had received extensive training in the standard MESA protocol; this is more close to the clinical scenario, in which a follow up exam is more likely to be performed by a different technician. Taking into consideration, the additional sources of variability, the lower level of agreement in the inter study analysis when compared with intra and inter reader analyses is understandable. The assessment of inter study variability is essential in the clinical scenario where the same exam is performed on a patient at different times to assess, for instance, the effect of one therapy.

### Limitations

The focus of CMR is most commonly the acquisition of LV images rather than LA images, as was the case in our study, resulting in some cases with poor LA image quality, in which it is challenging to accurately and reproducibly segment the LA both manually and by MTT. We had technical issues in 3 cases: i) Short axis image did not cover the entire LA; ii) Marked aorta overriding in four Chamber View and significant flow artifacts: iii) Bad slice orientation. Another limitation of this study is the relatively small number of subjects used for assessment of variability. While the sample size is typical for test-retest studies, we believe that the strength of the study could have improved further with a larger sample size.

## Conclusions

In conclusion, high spatial resolution MRI images provide for accurate LA chamber delineation, MTT derived biplane LA structure and function analysis is fairly accurate, less time consuming, reproducible and could potentially be a valuable tool for clinicians.
